# A Study on the Growth and Development Characteristics of Lindian Chickens

**DOI:** 10.3390/ani14020354

**Published:** 2024-01-22

**Authors:** Jie Liu, Yitong Jin, Shijie Zhou, Xinyu Wang, Yumao Li, Peng Luan, Hui Li, Li Leng, Yuxiang Wang

**Affiliations:** 1 Key Laboratory of Chicken Genetics and Breeding, Ministry of Agriculture and Rural Affairs, Harbin 150030, China; 2 Key Laboratory of Animal Genetics, Breeding and Reproduction, Education Department of Heilongjiang Province, Harbin 150030, China; 3 College of Animal Science and Technology, Northeast Agricultural University, Harbin 150030, China

**Keywords:** Lindian cockerels, body weight, carcass traits, internal organ traits, growth, correlations

## Abstract

**Simple Summary:**

Lindian chickens are a local chicken variety in northern China. To explore the growth and development characteristics of Lindian chickens, various statistical methods were used to analyze their body size and carcass traits. The results showed that multiple trait measurements of Lindian chickens rapidly increased before 10 weeks of age, while after 19 weeks of age they mainly developed reproductive organs and muscles. In addition, we also found that body size traits were closely related to carcass traits, providing a foundation for the breeding and management of Lindian chickens.

**Abstract:**

As an excellent chicken breed found in a high-altitude zone of northern China, Lindian chickens are characterized by good egg and meat production, strong adaptability, cold tolerance, rough feeding resistance, excellent egg quality, and delicious meat quality. To facilitate the exploitation of the unique qualities of the Lindian chicken, the varying patterns and correlations of various body size and carcass traits of 3–22-week-old Lindian chickens were analyzed in this study. The optimal growth model of these traits was determined by growth curve fitting analysis. The results showed that most traits of Lindian chickens increased steadily with increasing age, and most of them increased rapidly before 10 weeks of age. In addition, the inflection point age of each trait was predicted to be between 4 and 10 weeks. Furthermore, this study revealed that body size traits were closely related to carcass traits in Lindian chickens. In summary, Lindian chickens are in a rapid growth stage before the age of 10 weeks, and better slaughter performance can be achieved through good feeding management during this stage. The reproductive traits and muscles are the main developmental focus after the age of 19 weeks, so it is important to adequately meet their energy requirements for subsequent good breeding performance.

## 1. Introduction

China’s broiler industry is mainly divided into two categories: white-feather broilers and yellow-feather broilers. The market proportion of white-feather broilers is higher than that of yellow-feather broilers. White-feather broilers are mainly distributed in the northern area of the Yangtze River, with fast food consumption as the main focus. Yellow-feathered broilers meet the tastes of residents in the Yangtze River basin and southern areas with their advantages of taste and flavor [1]. According to their growth rate and time-to-market, yellow-feather broilers can be divided into three types: fast, medium, and slow (high quality). Among them, the fast type has a slaughter time of approximately 65 days and a low feed–weight gain ratio and is suitable for large-scale slaughter; the medium type has a slaughter time of 65–95 days, with growth speed and meat quality in the middle; and the slow type has a slaughter time of more than 95 days, superior meat quality, and higher prices [2].

The body composition traits (body size, carcass) of poultry can reflect its body form and condition and are important phenotypic traits. Growth traits can reflect the quality of poultry growth and development and the level of feeding management, and carcass traits can reflect the slaughtering performance and meat production performance of broilers. However, it is not easy to directly measure carcass traits during actual production. Therefore, the relationship between body form and traits is often utilized for indirect determination and early selection of these traits to assist breeding [3,4]. To explore the pattern of the growth and development of poultry, carry out breeding engineering reasonably, and achieve genetic improvement of economic traits, researchers often use statistical methods such as growth curve and correlation analysis to study the internal connection between traits during breeding and simplify many traits with complex relationships to improve breeding efficiency [5,6,7,8].

Intensive farming mainly involves the hybridization of pure commercial breeds with a high production performance, providing humans with a large amount of meat [9]. However, in Asia, Africa, South America, and the South Pacific region, local chicken populations still made significant contributions to the local economy [10,11]. As is well-known, local chickens have ideal characteristics, such as resistance to certain diseases and outstanding meat flavor and taste [12,13]. Researchers believed that although the growth performance of local chickens was not as good as commercial chickens, their meat quality was better [14]. The Lindian chicken, with a time-to-market of approximately 150 days of age, belongs to the slow-type yellow-feather broilers. It is one of China’s excellent local chicken breeds, characterized by good egg and meat production, strong adaptability, cold resistance, rough feeding resistance, excellent egg quality, and delicious meat quality, and it has been listed on the “National Animal Genetic Resources Protection List of China” since 2014 [15]. To facilitate the exploitation of the unique qualities of Lindian chickens, multiple scholars have conducted research on their genetic characteristics, but there are few reports on the growth and development characteristics of Lindian chickens [16,17,18]. Therefore, several methods were utilized in this study, such as correlation analysis and growth curve fitting, to comprehensively analyze the body composition traits of Lindian chickens at different developmental stages. The purpose was to explore the changes in various traits during the growth process of Lindian chickens, summarize their growth and development characteristics, and provide a theoretical basis for the breeding and conservation of yellow-feather broilers.

## 2. Materials and Methods

### 2.1. Experimental Animals

The Lindian chickens in this study came from the poultry breeding farm of Northeast Agricultural University, and a total of 8000 Lindian chickens, a protection resource population, were raised in this farm. All experimental chickens were housed in an environmentally controlled room. The chickens were vaccinated and wing-tagged at hatching. The chickens were kept in metal cages equipped with a row of nipple waterers, and a hanging feeder. The chickens and environmental controls were checked twice daily by trained staff. At different life stages, the chickens were fed different diets and housed in different-sized metal cages. During the rearing period, the chickens had ad libitum access to feed and water. At 0–2 weeks of age, the diet contained CP (Crude Protein), 20%, and ME (Metabolizable Energy), 3000 Kcal/kg; from 3 to 6 weeks of age, the diet contained CP 18% and ME 2900 Kcal/kg; from 7 to 15 weeks of age, the diet contained CP 16% and ME 2800 Kcal/kg; and from 16 to 22 weeks of age, the diet contained CP 16% and ME 2700 Kcal/kg. An amount of 29–42 cockerels were randomly selected at the ages of 3 weeks old, 6 weeks old, 10 weeks old, 14 weeks old, 19 weeks old, and 22 weeks old in the present experiment, with the total number of cockerels selected being 231.

### 2.2. Measurement Indicators and Methods

The body size and carcass traits of Lindian chickens were measured at the above six time points. Body size traits included body weight, body slope length (cm), sternum length (cm), breast width (mm), breast angle (°), tibia length (mm), and tibia circumference (mm). Carcass traits included heart weight (g), liver weight (g), gizzard weight (g), proventriculus weight (g), testis weight (g), and abdominal fat weight (g). Internal organs’ content (%) = internal organs’ weight (heart, liver, gizzard, proventriculus, and testis)/body weight × 100. Abdominal fat percentage (%) = abdominal fat weight/body weight × 100. The body slope length, sternum length, and tibia length were measured by tape. The breast width and tibia length were measured by vernier caliper. The body weight and internal organs were measured by electronic scales. The measurement methods of the above indicators were carried out in accordance with the standard “Terminology and Statistical Methods for Measurement of Poultry Production Performance” (NY/T 823-2004) [19].

### 2.3. Data Statistical Analysis

MS Excel 2016 was used to organize the data and establish a database to calculate the average and standard deviation of each measurement and the cumulative growth coefficient and relative growth coefficient. The cumulative growth coefficient is the actual measured value of a certain trait at each age stage, and the relative growth coefficient, (%) = [2 × (final value − initial value)/(final value + initial value)] × 100, is used to express the growth intensity of livestock and poultry, that is, the degree of growth at a certain stage. IBM SPSS statistical s27 was used to conduct one-way ANOVA, multiple comparison, correlation analysis, and growth curve fitting. Logistic, Gompertz, and Bertalanffy models were used to fit and analyze the body composition traits. The growth curve fitting model expressions and parameters are shown in Table 1. The results are expressed as the mean ± standard deviation. *p* < 0.05 indicates a significant difference, and *p* < 0.01 indicates that the difference is extremely significant.

## 3. Results

### 3.1. Patterns of Change in Body Composition Traits of Lindian Chickens

#### 3.1.1. Comparative Analysis of Body Composition Traits

**Figure 1:**
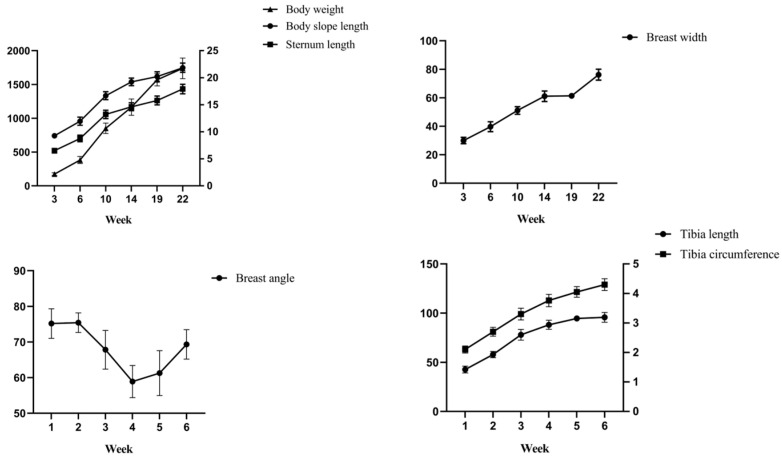
Trend of changes in body size traits.

**Figure 2:**
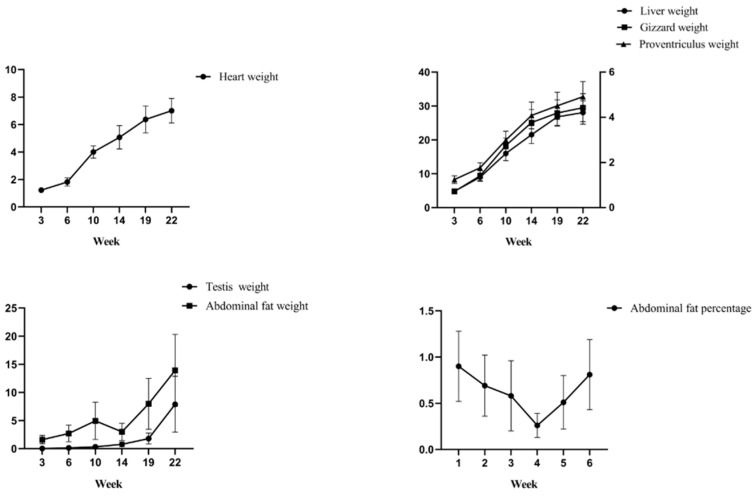
Trend of changes in carcass traits.

To determine the changes in various body composition traits of Lindian chickens during the rearing period, six stages (3 weeks old, 6 weeks old, 10 weeks old, 14 weeks old, 19 weeks old, and 22 weeks old) were selected from birth to market age, seven body size indicators and six carcass indicators were measured, and one-way ANOVA and multiple analysis were performed on the above traits at each time point. The results of the body size traits showed that with increasing age, body weight, body slope length, sternum length, and tibia circumference increased extremely significantly (*p* < 0.01), breast width and tibia length showed an upwards trend, but breast angle had no obvious trends (Table 2, Figure 1). The results of the carcass traits showed that with increasing age, the heart weight and testis weight increased extremely significantly (*p* < 0.01), and the liver weight, gizzard weight, and proventriculus weight showed an overall upward trend (Table 3, Figure 2). Furthermore, abdominal fat weight increased extremely significantly at 3–10 weeks (*p* < 0.01), decreased at 14 weeks, and increased again after 14 weeks of age (*p* < 0.01). The results showed that with increasing age, heart content showed an overall downward trend, with a significant difference at 3–6 weeks (*p* < 0.01). Liver content, gizzard content, and proventriculus content decreased significantly at 3–10 weeks and at 14–22 weeks (*p* < 0.05 or *p* < 0.01). Testis content increased extremely significantly at 3–6 weeks and at 10–22 weeks (*p* < 0.01). Abdominal fat percentage decreased before 14 weeks of age and increased significantly at 14 to 22 weeks of age (*p* < 0.01).

#### 3.1.2. Growth Intensity Analysis of Body Composition Traits

**Figure 3:**
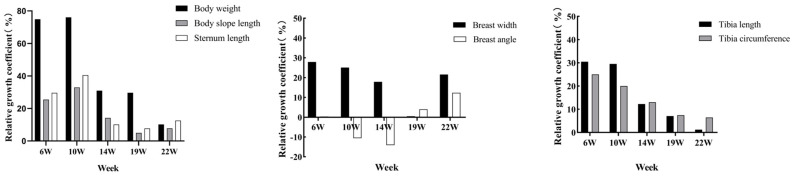
Relative growth coefficients of body size traits.

**Figure 4:**
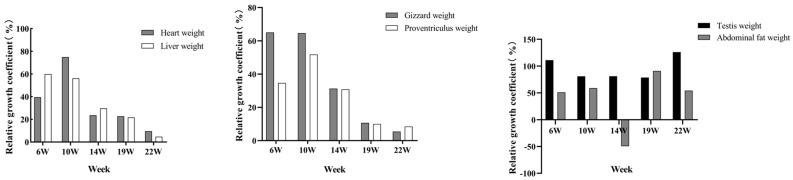
Relative growth coefficients of carcass traits.

To explore the growth intensity of Lindian chickens at each growth stage, the relative growth coefficients of body composition traits were calculated. The results of the body size trait measurements showed that the growth intensity of body weight, body slope length, sternum length, tibia length, and tibia circumference increased before 10 weeks of age and then showed a downwards trend (Figure 3). The growth intensity of body weight, body slope length, and sternum length was the highest at 10 weeks of age, reaching 76.05%, 32.96%, and 40.51%, respectively; the growth intensity of breast width, tibia length, and tibia circumference were the highest at 6 weeks of age, at 27.91%, 30.48%, and 25.00%, respectively; and the growth intensity of the breast angle was the highest at 22 weeks old, reaching 12.33%. The results of the carcass traits showed that heart weight, proventriculus weight, and gizzard weight increased before 10 weeks of age and then showed a downwards trend (Figure 4). The growth intensity of heart weight and proventriculus weight reached a maximum at 10 weeks of age, at 74.91% and 51.79%, respectively; the growth intensity of liver weight and gizzard weight was the highest at 6 weeks old, at 59.90% and 65.06%, respectively; the testis weight had the highest growth intensity at 22 weeks of age, reaching 126.03%; and the highest growth intensity of abdominal fat was at 19 weeks of age, reaching 90.98%.

### 3.2. Curve Fitting of Growth Traits in Lindian Chickens

**Figure 5:**
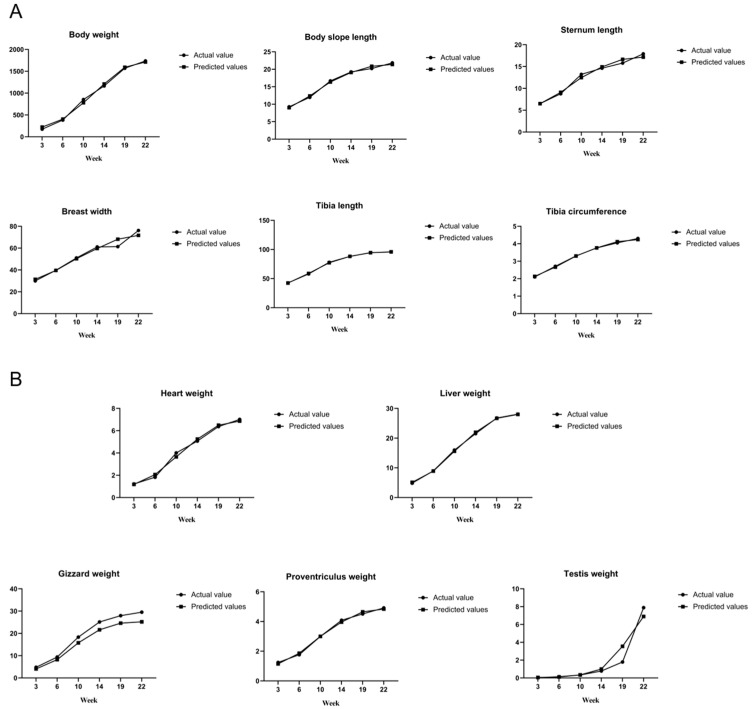
Curve fitting of body composition traits. (**A**): Fit graph of growth curve for body size traits. (**B**): Fit graph of growth curve for carcass traits.

To further explore the development patterns of different traits of Lindian chickens, three growth curve models, Gompertz, logistic, and Bertalanffy, were used in this study to fit the body composition traits and estimate the parameters of the growth model, the amount of growth at the inflection point, and the inflection point age. After comparing the fitting results of the models for various traits, the logistic model was ultimately identified as the optimal fitting model for each trait. The results indicated (Table 4 and Figure 5) that the body size traits of multiple individuals in Lindian chickens had achieved a high degree of fit, and the inflection point growth amounts of body weight, body slope length, sternum length, breast width, tibia length, and tibia circumference were 923.15 g, 11.01 cm, 8.91 cm, 39.83 mm, 48.77 mm, and 2.23 cm, respectively. The inflection point ages of body weight, body slope length, sternum length, breast width, tibia length, and tibia circumference were 11.32, 4.80, 5.79, 6.09, 4.17, and 3.59, respectively. Among the carcass traits (Table 5), the inflection point growth amounts of heart weight, liver weight, gizzard weight, proventriculus weight, and testis weight were 3.63 g, 14.69 g, 12.78 g, 2.53 g, and 13.14 g, respectively. The inflection point ages of body weight, heart weight, liver weight, gizzard weight, proventriculus weight, and testis weight were 9.96, 9.49, 8.45, 8.35, and 25.75, respectively.

### 3.3. Correlation Analysis of Growth Traits of Lindian Chickens

To explore the connection between the body composition traits of Lindian chickens, a correlation analysis was conducted between the above body composition traits. During the analysis process, partial correlation analysis was performed to control the time variable factor. The results are shown in Table 6. Among the body size traits, body weight, body slope length, sternum length, breast width tibia length, and tibia circumference were extremely significantly positively correlated with each other (*p* < 0.01); breast angle was significantly or extremely negatively correlated with body slope length, sternum length, tibia length, and tibia circumference (*p* < 0.05 or *p* < 0.01). There was no significant correlation between breast width and breast angle (*p* > 0.05).

Among the carcass traits, liver weight, gizzard weight, and proventriculus weight were extremely significantly positively correlated with each other (*p* < 0.01); testis weight was extremely significantly negatively correlated with body weight, liver weight, gizzard weight, and proventriculus weight (*p* < 0.01); there was an extremely significant positive correlation between heart weight and gizzard weight and proventriculus weight (*p* < 0.01); there was an extremely significant positive correlation between abdominal fat weight and testis weight (*p* < 0.01); and abdominal fat percentage was extremely significantly negatively correlated with the gizzard weight and extremely significantly positively correlated with the testis weight (*p* < 0.01).

Taken together, these results indicated that body slope length was extremely significantly positively correlated with body weight, heart weight, liver weight, gizzard weight, and proventriculus weight, and negatively correlated with testis weight and abdominal fat percentage (*p* < 0.01); sternum length had a significant negative correlation with testis weight (*p* < 0.05), and an extremely significantly positive correlation with other carcass traits (*p* < 0.01), but no significant correlation with abdominal fat traits; breast width was extremely significantly positively correlated with body weight, heart weight, gizzard weight, and proventriculus weight (*p* < 0.01); breast angle was extremely significantly positively correlated with testis weight, abdominal fat weight, and abdominal fat percentage (*p* < 0.01), and was significantly or extremely significantly negatively correlated with other carcass traits (*p* < 0.05 or *p* < 0.01), but had no significant correlation with heart weight; tibia length was extremely significantly negatively correlated with testis weight, abdominal fat weight, and abdominal fat percentage, and extremely significantly positively correlated with other carcass traits (*p* < 0.01); and tibia circumference was extremely significantly positively correlated with heart weight, liver weight, gizzard weight, and proventriculus weight (*p* < 0.01), and significantly negatively correlated with abdominal fat percentage *(p* < 0.05).

## 4. Discussion

In the production and breeding of livestock and poultry, some important quantitative traits can be difficult to directly measure. Therefore, researchers often achieve indirect or early selection of these traits by determining phenotypic traits such as body shape and weight that are closely related to them. Analyzing the correlation between the same trait and different developmental stages and different traits at the same developmental stage can provide a reference for early prediction and indirect selection. Based on this, the aim of the present study was to determine the overall change patterns and growth rates of various body composition traits and speculate on the important stages of growth and development of Lindian chickens by comparing and analyzing the changes in the same trait at different developmental stages and various traits at different growth and development stages, measuring growth intensity, and fitting growth curves. Furthermore, the correlation between traits was analyzed to select the main interrelated traits and their appropriate feeding management methods to provide a reference for trait selection and breeding.

Body size and body weight can directly or indirectly reflect the physical appearance and production performance of poultry and are important indicators for measuring the growth and development of poultry. At present, research on the body size traits of poultry is mostly focused on fixed time points, and there is very little research on the entire growth cycle. In this study, the body weight, body slope length, sternum length, breast width, tibia length, and tibia circumference of Lindian chickens showed an increasing trend with increasing age, which was similar to the results of Li et al. on a hybrid variety (Gushi chicken and Anka chicken) [20]. The breast angle showed a fluctuating variation in general but increased at 3–6 weeks of age, which was similar to the results of Ma et al. on AA chickens [21]. The steady increase in body size traits reflected that Lindian chickens had been in a relatively good growth and development state before they were marketed at 22 weeks of age. Carcass traits are important indicators for measuring the slaughter performance of poultry. The results of carcass traits showed that heart weight, liver weight, gizzard weight, proventriculus weight, and testis weight increased with age, which was consistent with some previous research findings. Ning et al. showed that the heart weight, liver weight, gizzard weight, and proventriculus weight increased with age in Qingjiao broiler chickens [22]. In addition, in this study the visceral content showed a decreasing trend. Lan et al. showed that liver content decreased with age, while liver weight and abdominal fat weight increased in yellow-feathered broilers [23]. The testis content showed an increasing trend; this is similar to the results of Mfoundou et al. [24]. However, the variation trend of abdominal fat traits was different from that of previous studies. A study on Beijing You chickens showed that the abdominal fat weight increased from 0 to 20 weeks of age and then decreased. The percentage of abdominal fat decreases at the age of 6 weeks and then increases over time [25]. Xie et al. showed that abdominal fat weight and the percentage of abdominal fat increased at 16, 18, and 20 weeks of age in a study of Qingyuan Partridge chickens [26]. In the present study, the weight of abdominal fat first increased, then decreased, and then increased, with the abdominal fat percentage first decreasing and then increasing. The increase in abdominal fat percentage in Lindian chickens in the later stage may be related to the need to accumulate energy and fat for the start of production. Overall, the majority of body composition traits of Lindian chickens had similar growth patterns to those of other breeds, with slight differences in breast angle and abdominal fat traits. This difference to some extent reflects the unique characteristics of breast development, immune function, and abdominal fat deposition in Lindian chickens.

When exploring the growth intensity of body size traits of Lindian chickens during the breeding period, this study showed that the growth intensity of body weight, body slope length, and sternum length was the highest at 10 weeks of age; the growth intensity of tibia length, tibia circumference, and breast width was the highest at 6 weeks of age; and the growth intensity of the breast angle was the highest at 22 weeks of age. In carcass traits, heart weight, liver weight, gizzard weight, and proventriculus weight reached their maximum growth intensity at 10 weeks of age and showed an overall downwards trend; the testis weight decreased before 19 weeks of age and reached its maximum growth intensity at 22 weeks of age; and the growth intensity was the highest at 19 weeks of abdominal fat weight. The above results indicated that the rapid growth stage of most traits in Lindian chickens was mainly concentrated before the age of 10 weeks, manifested in the development of body shape and internal organs. Ono et al. showed that the growth trend of tibia length is slow after 11 weeks of age [27]. However, Udoumoh et al. found that after sexual maturity the weight and volume of the testis were significantly greater than before sexual maturity, indicating that the testicles were developing late [28]. This is consistent with the discovery in this study that the testis weight of Lindian chickens reached its maximum growth rate in the later stage, implying that Lindian chickens reached sexual maturity relatively late. In general, during the growth and development process of Lindian chickens, traits such as body slope length, tibia length, and breast width mainly developed before 10 weeks of age, indicating that bone development was the main focus at this stage, while muscle, fat, and reproductive organs mainly developed after 19 weeks of age.

The growth curve is a mathematical model established to describe the growth of an animal’s body weight or an organ over time. The commonly used fitting models for predicting growth patterns in poultry are logistic, Gompertz, and Bertalanffy [29]. Different varieties and different traits of the same variety are suitable for different fitting models, so the model suitable for the studied variety should be the main choice in actual production. For example, Nguyen et al. identified Gompertz as the best fitting model for body weight when exploring the optimal growth model for local chickens in Vietnam [30]. Neysi et al. used eight models to fit the growth characteristics of Fars indigenous chickens, and ultimately determined that the Gompertz model is more suitable for describing its growth pattern [31]. Manjula et al. found that the logistic model was more suitable for fitting the growth of Korean native chickens [32]. Based on the results of this study, we found that logistic regression was more suitable for application in Lindian chickens. The growth curve model can reflect the growth characteristics of poultry. By fitting the growth curve of Wumeng chickens, Bai et al. found that the inflection point week age of the body weight was 3.49, and the inflection point weight was 753.84 g. The age of the tibia length inflection point was 2.32 weeks, and the tibia length at the inflection point was 4.26 cm [33]. Mignon et al. fitted the weight of chickens of different genders and found that the inflection point age of the males was around 9 weeks, with an inflection point weight of 1191 g, and it was higher than that of females [34]. The fitting results of the growth curve in this study indicated that the inflection point age of the body weight of Lindian chickens was approximately 12 weeks, which was later compared to other breeds, indicating that the development of Lindian chickens was slower. The inflection points of body slope length and sternum length were at approximately 5 and 6 weeks old, respectively, and their growth intensity was the highest at 6–10 weeks old, indicating that it is important to focus on feeding management and nutritional needs at this stage to meet their energy needs for development. The inflection points of tibia length, tibia circumference, and breast width were at approximately 5 weeks old, 4 weeks old, and 6 weeks old, respectively, and their growth intensity was at its highest at 3–6 weeks old. This stage is the main developmental stage for the above traits, and targeted breeding and selection for these traits can be carried out in this period. In carcass traits, the inflection point ages of the heart, liver, gizzard, and proventriculus were approximately 9 and 10 weeks, and their growth intensity reached its maximum at 10 weeks, indicating that 6–10 weeks is the main developmental period for visceral organs. The testicle was predicted to be at its inflection point at 25 weeks, and its growth intensity reached its maximum at 22 weeks, indicating that the testicle continued to grow rapidly after 22 weeks, and after this stage Lindian chickens began to prepare for breeding. Compared with other breeds, the inflection point of body weight and body size traits was later, indicating that the early growth and development of Lindian chickens may be relatively slow. In summary, to ensure the good development of body weight, some body size traits (body slope length, tibia length, and tibia circumference), and carcass traits in Lindian chickens, it is necessary to strengthen feeding management between 4 and 10 weeks of age, and after 22 weeks of age attention should be given to reproductive traits more closely.

Body size and carcass traits can reflect the production performance of livestock and poultry, and research on them can provide a theoretical reference for selecting excellent livestock and poultry varieties. The results of the correlation analysis between body size and carcass traits of Lindian chickens in this study indicated that most body size traits had significant correlations with each other. Brito et al. also showed a highly positive correlation between weight and body size traits; this indicated that the development of Lindian chickens was relatively coordinated during growth and development [35]. Among them, there was a high correlation between body weight, body slope length, sternum length, breast width, tibia length, and tibia circumference, which was similar to the results for Xiayan chickens [36]. However, the results of Xiaoshan chickens showed no significant correlation between these traits, indicating that there was a difference in body shape between Lindian chickens and this breed [37]. The traits with a high correlation between carcass traits in Lindian chickens mainly include liver weight, gizzard weight, and proventriculus weight. Gaya et al. found a negative correlation between liver and gizzard, with a relatively low degree of correlation, indicating that there were differences in the growth and development patterns between Lindian chickens and this breed [38]. In addition, there was a significant correlation between most body size traits and carcass traits in Lindian chickens. Yang et al. showed that abdominal fat weight was negatively correlated with body slope length, sternum length, and tibia circumference, and was significantly negatively correlated with tibia length, which was consistent with this study [39]. Lang et al. found that breast width, body slope length, and tibia length can be used as indirect breeding indicators for abdominal fat deposition traits [40]. In this study, abdominal fat traits were mainly correlated with chest width, tibia length, and testicular weight, which combined with the changing pattern of the abdominal fat traits of Lindian chickens allows us to hypothesize that the fat deposition of Lindian chickens may have a unique pattern. According to the above results, in the breeding process of Lindian chickens body weight and visceral organ development can be predicted based on body slope length, sternum length, and tibia length. Furthermore, abdominal fat deposition can be indirectly tracked by monitoring changes in breast width, tibia length, and testis weight in Lindian chickens.

## 5. Conclusions

In the growth stage, from 3 to 22 weeks of age, most of the body size and carcass traits of Lindian chickens increased steadily with increasing age, and the rapid growth stage of most traits was before 10 weeks of age. Furthermore, logistic regression was identified as the optimal growth model to describe its various traits, and the inflection point age of these traits was predicted to be between 4 and 10 weeks. In addition, this study revealed a high degree of correlation between body slope length, sternum length, tibia length, body weight, liver weight, gizzard weight, and proventriculus weight.

## Figures and Tables

**Table 1:** Three commonly used growth curve models for livestock and poultry.

Model	Expression	Inflection Point Length/Weight	Inflection Point Age
Logistic	Y = A/(1 + Be^−kt^)	A/2	(lnB)/K
Gompertz	Y = Ae^−Bexp(−kt)^	A/e	(lnB)/K
Bertalanffy	Y = A(1 − Be^−kt^)^3^	8A/27	(ln3B)/K

Note: Y is the predicted value; A is the limiting growth; B is a constant parameter; k is the instantaneous relative growth rate; and t is the age parameter.

**Table 2:** Comparative analysis of body size traits at different ages.

Body Size Traits	3 Weeks	6 Weeks	10 Weeks	14 Weeks	19 Weeks	22 Weeks
	*n* = 29	*n* = 40	*n* = 39	*n* = 39	*n* = 42	*n* = 42
Body weight (g)	174.10 ± 20.18 ^F^	382.48 ± 52.18 ^E^	851.82 ± 77.03 ^D^	1164.26 ± 119.79 ^C^	1569.76 ± 90.01 ^B^	1738.07 ± 152.09 ^A^
Body slope length (cm)	9.26 ± 0.40 ^F^	11.96 ± 0.76 ^E^	16.68 ± 0.73 ^D^	19.23 ± 0.70 ^C^	20.21 ± 0.88 ^B^	21.84 ± 0.86 ^A^
Sternum length (cm)	6.50 ± 0.41 ^F^	8.76 ± 0.60 ^E^	13.21 ± 0.78 ^D^	14.62 ± 0.73 ^C^	15.79 ± 0.82 ^B^	17.91 ± 0.88 ^A^
Breast width (mm)	29.96 ± 2.26 ^E^	39.68 ± 3.46 ^D^	51.06 ± 2.70 ^C^	61.08 ± 3.68 ^B^	61.36 ± 3.74 ^B^	76.18 ± 3.84 ^A^
Breast angle (°)	75.17 ± 4.15 ^A^	75.42 ± 2.75 ^A^	67.82 ± 5.44 ^B^	58.88 ± 4.50 ^C^	61.26 ± 6.30 ^C^	69.31 ± 4.13 ^B^
Tibia length (mm)	42.57 ± 3.45 ^E^	57.88 ± 3.19 ^D^	77.94 ± 5.48 ^C^	88.12 ± 4.64 ^B^	94.56 ± 2.96 ^A^	95.70 ± 4.95 ^A^
Tibia circumference (cm)	2.10 ± 0.12 ^F^	2.70 ± 0.15 ^E^	3.30 ± 0.20 ^D^	3.76 ± 0.21 ^C^	4.05 ± 0.18 ^B^	4.30 ± 0.20 ^A^

Note: Different capital letters in the same row indicate extremely significant differences (*p* < 0.01).

**Table 3:** Comparative analysis of carcass traits at different ages.

Carcass Traits	3 Weeks	6 Weeks	10 Weeks	14 Weeks	19 Weeks	22 Weeks
	*n* = 29	*n* = 40	*n* = 39	*n* = 39	*n* = 42	*n* = 42
Heart weight (g)	1.22 ± 0.16 ^F^	1.82 ± 0.29 ^E^	4.00 ± 0.44 ^D^	5.07 ± 0.85 ^C^	6.37 ± 0.98 ^B^	7.01 ± 0.89 ^A^
Liver weight (g)	4.83 ± 0.49 ^E^	8.96 ± 1.17 ^D^	15.96 ± 2.14 ^C^	21.52 ± 2.65 ^B^	26.76 ± 2.53 ^A^	28.05 ± 3.44 ^A^
Gizzard weight (g)	4.77 ± 0.76 ^Ee^	9.37 ± 1.47 ^Dd^	18.31 ± 2.27 ^Cc^	25.10 ± 3.85 ^Bb^	27.94 ± 3.88 ^ABa^	29.50 ± 4.10 ^Aa^
Proventriculus weight (g)	1.24 ± 0.17 ^Ee^	1.76 ± 0.23 ^Dd^	2.99 ± 0.3 ^Cc^	4.08 ± 0.59 ^Bb^	4.51 ± 0.60 ^ABa^	4.91 ± 0.67 ^Aa^
Testis weight (g)	0.04 ± 0.02 ^F^	0.14 ± 0.04 ^E^	0.33 ± 0.13 ^D^	0.78 ± 0.42 ^C^	1.79 ± 0.97 ^B^	7.89 ± 4.96 ^A^
Abdominal fat weight (g)	1.60 ± 0.73 ^Ee^	2.70 ± 1.49 ^Dd^	4.95 ± 3.31 ^BCc^	2.99 ± 1.54 ^CDd^	7.98 ± 4.53 ^Bb^	13.92 ± 6.40 ^Aa^
Heart content (%)	0.70 ± 0.07 ^Aa^	0.48 ± 0.06 ^Bb^	0.47 ± 0.05 ^BCc^	0.43 ± 0.06 ^BCc^	0.41 ± 0.06 ^Cc^	0.40 ± 0.04 ^Cc^
Liver content (%)	2.79 ± 0.24 ^Aa^	2.36 ± 0.27 ^Bb^	1.87 ± 0.19 ^Cc^	1.85 ± 0.21 ^CDc^	1.71 ± 0.18 ^Dd^	1.62 ± 0.18 ^Ee^
Gizzard content (%)	2.74 ± 0.31 ^A^	2.46 ± 0.33 ^B^	2.16 ± 0.28 ^C^	2.16 ± 0.30 ^C^	1.78 ± 0.25 ^D^	1.70 ± 0.18 ^D^
Proventriculus content (%)	0.71 ± 0.08 ^A^	0.46 ± 0.05 ^B^	0.35 ± 0.04 ^C^	0.35 ± 0.05 ^C^	0.29 ± 0.04 ^D^	0.28 ± 0.03 ^D^
Testis content (%)	0.03 ± 0.01 ^Aa^	0.04 ± 0.01 ^Bb^	0.04 ± 0.02 ^Bc^	0.07 ± 0.04 ^Cd^	0.12 ± 0.06 ^Cd^	0.45 ± 0.28 ^De^
Abdominal fat percentage (%)	0.90 ± 0.38 ^Aa^	0.69 ± 0.33 ^ABabc^	0.58 ± 0.38 ^ABbc^	0.26 ± 0.13 ^Cd^	0.51 ± 0.29 ^Bc^	0.81 ± 0.38 ^Aab^

Note: Different lowercase letters in the same row indicate significant differences (*p* < 0.05), while different uppercase letters indicate extremely significant differences (*p* < 0.01).

**Table 4:** Fitting analysis of the growth curve of body size traits of Lindian chickens.

Traits	Model	A	B	K	Fitting (R^2^)	Inflection Point Growth Amount	Inflection Point Age (Weeks)
Body weight (g)	Logistic	1846.301	14.971	0.239	0.966	923.15	11.32
Body slope length (cm)	Logistic	22.020	2.66	0.204	0.963	11.01	4.80
Sternum length (cm)	Logistic	17.827	3.187	0.200	0.942	8.91	5.79
Breast width (mm)	Logistic	79.659	2.318	0.138	0.892	39.83	6.09
Tibia length (mm)	Logistic	97.545	2.601	0.229	0.952	48.77	4.17
Tibia circumference (mm)	Logistic	4.453	1.772	0.162	0.997	2.23	3.59

**Table 5:** Fitting analysis of the growth curve of carcass traits of Lindian chickens.

Traits	Model	A	B	K	Fitting (R^2^)	Inflection Point Growth Amount	Inflection Point Age (Weeks)
Heart weight (g)	Logistic	7.267	10.383	0.235	0.991	3.63	9.96
Liver weight (g)	Logistic	29.386	9.746	0.240	0.999	14.69	9.49
Gizzard weight (g)	Logistic	25.569	13.260	0.306	0.999	12.78	8.45
Proventriculus weight (g)	Logistic	5.052	6.770	0.229	0.995	2.53	8.35
Testis weight (g)	Logistic	26.274	1190.978	0.275	0.911	13.14	25.75

**Table 6:** Correlation analysis between growth traits of Lindian chickens.

	Body Slope Length	Sternum Length	Breast Width	Breast Angle	Tibia Length	Tibia Circumference	Body Weight	Heart Weight	Liver Weight	Gizzard Weight	Proventriculus Weight	Testis Weight	Abdominal Fat Weight	Abdominal Fat Percentage
Body slope length	1													
Sternum length	0.784 **	1												
Breast width	0.597 **	0.600 **	1											
Breast angle	−0.403 **	−0.210 *	0.036	1										
Tibia length	0.779 **	0.652 **	0.463 **	−0.488 **	1									
Tibia circumference	0.674 **	0.588 **	0.553 **	−0.235 **	0.627 **	1								
Body weight	0.561 **	0.411 **	0.298 **	−0.186 *	0.428 **	0.523 **	1							
Heart weight	0.379 **	0.315 **	0.236 **	−0.075	0.350 **	0.423 **	0.602 **	1						
Liver weight	0.460 **	0.328 **	0.123	−0.249 **	0.411 **	0.396 **	0.623 **	0.157	1					
Gizzard weight	0.590 **	0.436 **	0.281 **	−0.316 **	0.535 **	0.436 **	0.613 **	0.312 **	0.591 **	1				
Proventriculus weight	0.506 **	0.356 **	0.278 **	−0.304 **	0.417 **	0.465 **	0.562 **	0.313 **	0.563 **	0.631 **	1			
Testis weight	−0.369 **	−0.208 *	−0.003	0.416 **	−0.434 **	−0.127	−0.216 **	0.094	−0.486 **	−0.482 **	−0.366 **	1		
Abdominal fat weight	−0.159	−0.046	−0.046	0.281 **	−0.234 **	−0.049	0.077	−0.052	0.134	−0.143	−0.061	0.287 **	1	
Abdominal fat percentage	−0.297 **	−0.151	−0.131	0.307 **	−0.346 **	−0.194 *	−0.046	−0.095	−0.007	−0.230 **	−0.143	0.270 **	0.824 **	1

Note: “*”indicates a significant correlation, “**”indicates an extremely significant correlation.

## Data Availability

The data presented in this study are available on request from the corresponding author.
